# Videothoracoscopic Bronchial Sleeve Resection for Non-Small Cell Lung Cancer

**DOI:** 10.7759/cureus.13686

**Published:** 2021-03-04

**Authors:** Ali Celik, Muhammet Sayan

**Affiliations:** 1 Department of Thoracic Surgery, Gazi University Faculty of Medicine, Ankara, TUR

**Keywords:** lung cancer, sleeve resection, lobectomy, video-assisted thoracic surgery

## Abstract

Nowadays, videothoracoscopic lobectomy is accepted as the standard treatment method in early-stage lung cancer. Sleeve lobectomy, which is an alternative to pneumonectomy in centrally located tumors or peribronchial tumor infiltration, was previously performed via thoracotomy, but it can be performed by video-assisted thoracoscopic surgery (VATS) in recent years. In this method, without compromising oncological principles, a more comfortable and less morbid postoperative process can be provided to the patient compared to thoracotomy. Here, we aimed the presenting two cases that underwent VATS sleeve lobectomy for non-small cell lung cancer and their results.

## Introduction

Sleeve lung resection, a parenchyma-sparing lung resection technique, has more advantages than pneumonectomy in terms of early and late mortality/morbidity [1]. Many studies have reported that, in the treatment of lung cancer, lung resections performed with minimally invasive techniques produce better long-term outcomes and have reduced early mortality and morbidity compared to thoracotomies. Video-assisted thoracoscopic surgery (VATS) lobectomy has been established in the literature as a standard treatment method for early-stage lung cancer after a 2007 study conducted by Swanson et al. [2]. Since then, VATS has become a popular alternative to thoracotomy in many thoracic surgery procedures and has been preferred in sleeve resections in recent years. In suitable cases, VATS sleeve lobectomies for centrally located tumors are more advantageous than those performed via thoracotomy. Here, we present two cases that underwent VATS bronchial sleeve lobectomy and mediastinal lymph node dissection for non-small cell lung cancer, and the clinical and oncological results from each case.

## Case presentation

Case 1

**Figure 1:**
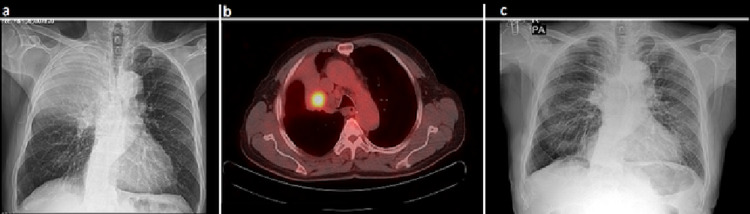
A chest x-ray showing the right upper lobe atelectasis (a), PET-CT showing a pathologically increased uptake of 18F-FDG on the mass (b), and the non-complicated chest x-ray taken at the discharge of the patient (c).

**Figure 2:**
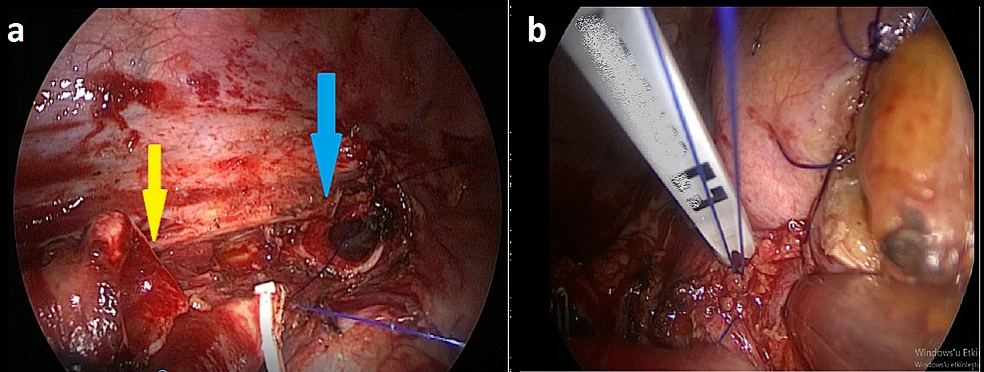
The yellow arrow indicates the right intermediate bronchus; the blue arrow indicates the right main bronchus (a) and the running suture technique uses a 3-0 polypropylene suture which is knotted outside of the bronchus (b).

A 75-year-old male patient was admitted to us with exertional dyspnea and cough complaints. Thoracic CT showed a right upper lobe collapse and a 25 mm diameter mass located in the right upper lobe bronchus (Figure 1a). A pathologically increased uptake of 18F-fluorodeoxyglucose (18F-FDG) (standardized uptake value {SUV} max: 5.5) was detected with PET-CT (Figure 1b). A mass obstructing the right upper lobe bronchus and extending into the main bronchus was detected in fiberoptic bronchoscopy (FOB) examination. The histopathological report from a bronchoscopic biopsy was of squamous cell carcinoma. There was no abnormality seen on a cranial magnetic resonance image, and a bi-portal VATS bronchial right upper sleeve lobectomy and a mediastinal lymph node dissection were performed. A running suture technique with 3-0 polypropylene suture material was used (Figures 2a, 2b). The patient’s postoperative period was uneventful, and he was discharged on the sixth postoperative day with a non-complicated chest roentgenogram (Figure 1c). The pathologic stage of the patient was pT2N1, and six cycles of cisplatin-based adjuvant chemotherapy were given to him. Follow-up of the patient found no local or distant recurrences after two years.

Case 2

**Figure 3:**
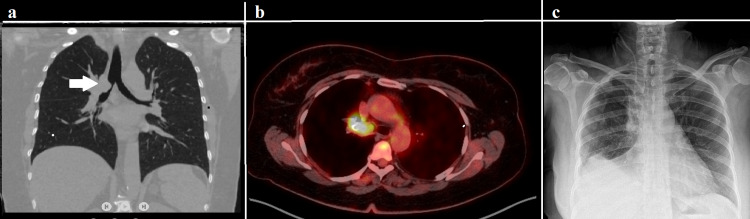
The chest CT showing a mass in the right upper lobe bronchus extending to the right main bronchus (arrow) (a); PET-CT showing a mass with high metabolic activity (b); x-ray, taken at discharge, showing the expansion of both lungs (c).

A 49-year-old female patient was admitted to us with cough complaints that had been ongoing for eight months. The thoracic CT showed a vegetant mass with a 3 x 2 diameter arising from the upper lobe bronchus and extending into the right main bronchus (Figure 3a). Histopathological examination of a biopsy taken via fiberoptic bronchoscopy revealed adenoid cystic carcinoma. Except for the tumor, no pathologically increased uptake of 18F-FDG was seen with PET-CT, and no lesions were detected with cranial MRI (Figure 3b). A VATS bronchial right upper sleeve lobectomy and a mediastinal lymph node dissection were performed. The surgical technique was similar to the first case. Frozen section examinations of the right main and intermediate bronchi were tumor-free. The pathologic stage of the patient was pT2N0 with complete resection (R0), and she was discharged on the seventh postoperative day. There were no complications seen in the chest radiography at discharge (Figure 3c). The patient was followed up in the sixth postoperative month without any recurrence.

## Discussion

In this report, we reviewed the current literature because our two cases underwent VATS bronchial sleeve lobectomies. The first VATS sleeve lobectomy reported in the literature was in 2002. In the following years, more complicated surgeries, such as uniportal VATS sleeve lobectomy and bronchovascular VATS sleeve lobectomy, were reported as case series [3]. In VATS sleeve lobectomies, the length of the incision is small, and an intercostal retractor is not used; thus, wound site problems, bleeding, pain, lung damage, hospital stay lengths, and air leakages are lessened in comparison to thoracotomies [1,4,5]. The disadvantage of VATS sleeve lobectomy is that the duration of the anastomosis is longer than thoracotomy. Zhou et al. and Wang et al. reported mean durations of anastomosis between 55 and 39 minutes, respectively [3,6]. In Ceylan et al. the reported range of anastomosis time was 55-110 minutes [5]. In our study, the anastomosis times were 82 minutes in the first case and 65 minutes in the second. Anastomosis time is short in the running suture technique, but it risks anastomosis dehiscence due to bronchial tears at the suture site. We preferred the running suture technique in both cases. The duration of anastomosis in VATS sleeve lobectomy will likely shorten as the experience increases. The perioperative complications from VATS sleeve lobectomies are similar to those from thoracotomy. These are pneumonia, atelectasis, air leak, cardiac arrhythmia, and bleeding. It has been reported that the incidence of these complications ranges from 4.8% to 31% [3,7]. Ceylan et al. reported that, in their series of 12 cases, there was one case of myocardial infarction and one case of prolonged air leak [5]. Wang et al. reported that there were no complications in their series [6]. The complications of our cases were as follows: in the first case, minimal subcutaneous emphysema was detected but did not require intervention, and in the second case, partial atelectasis occurred, which improved with bronchoscopic secretion aspiration. There is not enough study on the long-term outcomes of VATS sleeve lobectomies performed for lung cancer. Zhou et al. compared sleeve lobectomy performed via VATS to thoracotomy and found no significant difference in terms of median survival between the groups [3]. Ceylan et al. reported a mean disease-free survival of 36.1 months and mean survival of 42.7 months in their short-term study [5]. Wang et al. did not give any information about survival in their study [6]. The comparison of survival in sleeve lobectomies performed via thoracotomy and VATS may be biased because VATS sleeve lobectomy is preferred in well-selected cases, whereas it is not preferred in technically challenging cases.

## Conclusions

VATS sleeve lobectomy can be preferred in indicated cases, such as endobronchial lesions or peribronchial lymph node infiltration. Although the technical details, perioperative results, and short-term survival rates are satisfactory, there is a need for controlled studies with larger case series to evaluate the long-term outcomes of the oncologic cases.
